# Relationship between plasma NGAL and serum creatinine is influenced by leucocytosis and neutrophilia in the critically ill

**DOI:** 10.1186/cc11785

**Published:** 2012-11-14

**Authors:** NJ Glassford, AG Schneider, G Eastwood, L Peck, H Young, S Xu, R Bellomo

**Affiliations:** 1 Austin Hospital, Melbourne, Australia; 2 Uppsala University, Uppsala, Sweden; 3 Monash University, Melbourne, Australia

## Background

NGAL appears to be a biomarker of acute kidney injury. It is produced by neutrophils and renal tubular cells. In healthy patients, plasma NGAL levels have been shown to vary with white blood cell concentrations. The predictive ability of NGAL to predict AKI is attenuated by leucocytosis in patients being treated with vancomycin. The relationship between NGAL and white cell indices in the critically ill is poorly understood. The aim is to explore the relationship between different measures of NGAL and white cell count (WCC) and neutrophil count (NC) in critically ill patients at risk of AKI.

## Methods

Patients with systemic inflammatory response syndrome and oliguria, or a creatinine rise >25 μmol/l, or both, in a tertiary referral university hospital ICU, within 48 hours of admission, were studied. We measured kidney-specific monomeric urinary NGAL (muNGAL), plasma (pNGAL) and urinary NGAL (ucNGAL) by commercial assay, and routine haematological and biochemical parameters. Institutional ethics approval was obtained.

## Results

We studied 84 patients (51.2% male, median age 66.5 (52 to 74)) with a median APACHE III score of 61 (45 to76). Fifty-one patients had WCC >10 × 10^9^/l, 58 had NC >7.5 × 10^9^/l. No useful correlations were demonstrated between white cell indices and any form of NGAL measurement. A stronger correlation existed between creatinine and sNGAL than urinary indices. The strength of correlation between creatinine and all measures of NGAL was greater in those patients with high white cell indices (Table 1); the increase was significant for sNGAL in those with elevated WCC (*r *= 0.27 in nonleucoytotic patients, *r *= 0.65 in leucocytotic patients, *P *< 0.05).

**Table 1:** Spearman correlation for inclusion creatinine

Group	*n*	pNGAL	ucNGAL	muNGAL
Undifferentiated	84	0.51	0.40	0.40
No leucocytosis (WCC <10 × 10^9^/l)	33	0.27	0.22	0.21
Leucocytosis (WCC >10 × 10^9^/l)	51	0.65	0.47	0.47
No neutrophilia (NC <7.5 × 10^9^/l)	26	0.40	0.33	0.32
Neutrophilia (NC >7.5 × 10^9^/l)	58	0.55	0.42	0.41

## Conclusion

The stronger correlation between creatinine and pNGAL suggests systemic factors may be more important than local factors in the pathophysiology of AKI. The increased strength of relationship in those with leukocytosis suggests NGAL may be a general index of illness severity. Multicentre and longitudinal studies are required to further increase our understanding of the natural history of NGAL release and the clinical utility of these tests.

